# Presenting information on regulation values improves the public’s sense of safety: Perceived mercury risk in fish and shellfish and its effects on consumption intention

**DOI:** 10.1371/journal.pone.0188758

**Published:** 2017-12-21

**Authors:** Michio Murakami, Mai Suzuki, Tomiko Yamaguchi

**Affiliations:** 1 Department of Health Risk Communication, Fukushima Medical University School of Medicine, Fukushima, Fukushima, Japan; 2 Research Support Group, International Christian University, Osawa, Mitaka, Tokyo, Japan; 3 College of Liberal Arts, International Christian University, Osawa, Mitaka, Tokyo, Japan; Southwest University, CHINA

## Abstract

Risk communication aims to promote health and understanding through information exchange; however, explanations regarding the basis of regulation values for the public are insufficient. Moreover, it is unclear how information presentation affects the public’s sense of safety and their consumption intentions. We first investigated the relationship between perception of mercury-risk in fish and shellfish and individual attributes and knowledge. We then examined how presenting information on regulation values and primary factors regarding perception affected sense of safety toward regulations and food-consumption intentions. An online survey was conducted with Japanese individuals (N = 1148). Respondents were randomly assigned to one of three groups based on the presentation level of regulation values. People who frequently consumed tuna had a high perception of dread risk of mercury. This suggests that the dread risk perception of mercury does not determine tuna-type consumption behavior; rather, individuals’ consumption behavior determines dread risk perception of mercury. Among those with high tuna-type consumption, those receiving information that a safety factor of 10 times had been considered showed a significantly greater sense of safety than did the group that was not presented with information on regulation values (odds ratio (95% confidence interval): 2.04 (1.18–3.53), *p* < 0.05). However, presentation of regulation values showed a weak but significantly positive correlation with excessive intake of tuna-type fish (odds ratio: 2.95 (0.93–9.32), *p* < 0.10). Presenting the information on regulation values increases sense of safety; however, it may also lead to excessive intake.

## Introduction

When food risks are obvious, individuals decide to consume or avoid high-risk foods based on society’s regulation values. However, in general, since various experts’ judgments can determine food regulation values, the public finds this decision-making process confusing [1]. In particular, distrust of regulation values can lead to excessive avoidance, social economic losses due to decreased consumption, and a decrease in well-being resulting from concerns about food safety [2–4]. Consequently, the importance of risk communication has grown [1].

The U.S. National Research Council defines risk communication as “an interactive process of exchange of information and opinion among individuals, groups, and institutions” and states that “risk communication is successful to the extent that it raises the level of understanding of relevant issues or actions and satisfies those involved that they are adequately informed within the limits of available knowledge” [5]. The Food and Agriculture Organization of the United Nations (FAO)/World Health Organization (WHO) states that “effective food safety risk communication is defined as the exchange of information and opinions among people about the risks and risk-related factors associated with food safety hazards and risks” and “the goals of food safety risk communication are to enable people to protect their health from food safety risks by providing information that enables them to make informed food safety decisions, to facilitate dialogue and understanding among all interested stakeholders, and to improve the overall effectiveness of the risk analysis process” [6]. While risk communication aims to promote health and understanding through information exchange, the public is unaware of how regulation values are determined. Additionally, although research has been conducted on risk perception, acceptance, and presentation methods [7–10], the impact of presenting this information on the public’s sense of safety and consumption intentions has not been examined.

Risk acceptance is governed by perceptions of risk, benefit, and trust [11], and risk perception is reported to be associated with sex, age, presence of a spouse/children, knowledge, and cultural worldview [9,10,12,13]. Therefore, it is expected that sense of safety regarding regulation values and food-consumption behavior intention (e.g., excessive avoidance and excessive intake) are associated with risk perception. In order to determine the effects of presenting information on regulation values on sense of safety and food consumption intention by individual characteristics and risk perception, we need to consider the subject, situations, and purposes of the information in terms of risk communication.

This study addressed the risk of mercury in fish and shellfish. Exposure to methylmercury affects the central nervous system and has an adverse impact on developing fetuses [14,15]; therefore, methylmercury risk from fish and shellfish is a global concern [16]. Japan has experienced historic pollution from Minamata disease; in 1973, 0.4 mg/kg was set as the provisional regulation value for total mercury in fish and shellfish. A previous study noted that among Minamata disease patients and others in Japan, this regulation level was set below a safety factor of 10 times the lowest observed adverse effect level (LOAEL). This was also supported by the FAO/WHO Joint Expert Committee on Food Additives (held in 1972); this value is nearly the same as the safety factor of 50 times no observed adverse effect level (NOAEL) for chronic toxicity of methylmercury in monkeys [17]. However, some specific types of fish and shellfish contain naturally derived mercury at a higher concentration than these regulation values [14]. Consequently, the Japanese government has excluded tuna-type fish (i.e., tuna, marlin, and bonito) from these regulations.

Since 2003 (revised in 2010), the Japanese government has provided advice for pregnant women on the number of times they could consume specific types of fish and shellfish (e.g., big-eye tuna once per week) [18]. The U.S. Food and Drug Administration and the U.S. Environmental Protection Agency (EPA) also provide recommended consumption for pregnant or breastfeeding women, by classifying types of fish into “best choices” (eat two to three servings a week), “good choices” (eat one serving a week), and “fish to avoid” [19]. The average Japanese intake of mercury is currently 0.0011 mg/kg body weight per week (within that, 84.2% from fish and shellfish), which is lower than the tolerable consumption amount of 0.002 mg/kg body weight per week. On the other hand, with international popularity of Japanese food, among Americans who like tuna and sushi, blood mercury levels may exceed the EPA’s reference concentration [20].

First, we examined the association between risk perception of mercury and knowledge and individual attributes including sex, age, beliefs regarding fish and shellfish, and consumption frequency, and identified these perceptions’ primary underlying factors. Subsequently, we quantitatively examined how presentation of information on regulation values and these perception factors affected sense of safety regarding regulation values for fish and shellfish and food-consumption intention. This novel study, using mercury in fish and shellfish as an example, is the first to examine the impact of presenting evidence for regulation values on people’s sense of safety and food-consumption intentions.

## Methods

### Participants

Ethical approval for the study was granted by the Fukushima Medical University Ethics Committee (approval number: 2566).

An online survey was conducted from December 2–4, 2015. Participants were individuals aged 20–69, from across Japan who had registered with the Intage Research Inc., one of the largest survey companies in Japan with 1.32 million panelists. In the online survey, the company set up target number of participants, grouped according to sex, age, and residential area. They then asked registered members to respond to the questionnaires until this target number of participants was achieved. The targets were selected for consistency with actual composition ratios for age (20s, 30s, 40s, 50s, 60s), sex, and residential area (10 areas). Elderly individuals aged 70 years or older, who may have been unfamiliar with online surveys, were excluded. The company also adjusted response time to “within a day” to collect well-balanced data from participants throughout the collection period to reduce selection bias. Based on information on regulation values, the company assigned one of three conditions to participants before the questionnaire (for details, see “question items”) to avoid potential self-selection bias.

There were no missing data in the surveys. Responses were excluded in the case of inconsistencies in sex and age in Intage Research Inc.’s registration information and responses (± 1-year age difference was accepted), short response time, and multiple responses from the same IP address. Intage Research Inc. managed panelist information such as confirming participants’ location by sending a postcard to the registered address and eliminating inappropriate panelists. Respondents were encouraged to answer the survey by awarding points that could be exchanged for cash, Internet points, or gift certificates. The characteristics and advantages of the online survey are described in previous reports [10,21].

In total, data of 1148 participants were collected: 367 did not receive information on regulation values (A1), 387 were presented evidence based on epidemiological study findings (A2), and 394 were presented evidence stating that, as per epidemiological studies, health impacts are 10 times less than LOAEL (A3).

### Question items

All the questionnaires were in Japanese. We first obtained participants’ demographic data including sex; age; occupation; presence of spouse, children, or grandchildren; whether they/their spouse was pregnant; educational background, a science course or a humanities course (5 choices: *science course*, *science course chosen from between science course and humanities course*, *neither*, *humanities course chosen from between science course and humanities course*, *or humanities course*); frequency of fish and shellfish consumption (including processed foods and whale meat, and excluding seaweed; 8 choices: *nearly every day*, *5–6 times per week*, *4 times per week*, *3 times per week*, *twice per week*, *once per week*, *less than once per week*, and *do not eat*); how often they currently eat tuna-type fish (tuna, big-eye tuna, and bonito; same 8 choices, as above); and general beliefs regarding fish and shellfish (good for health (health-view), delicious (taste-view), and reasonably priced (availability-view)), scored on a 5-point Likert scale ranging from 5 (*strongly agree*) to 1 (*do not agree at all*) (Table 1). These question items were selected because they were reported as factors of risk perception in previous studies [9,10,12,13]. We obtained information on consumption frequency and general beliefs regarding fish and shellfish, because we hypothesized that risk perception would be associated with these daily everyday behaviors and beliefs.

**Table 1:** Respondents’ demographics, consumption frequency, beliefs about fish and shellfish, and knowledge. SD: standard deviation.

	N (%) or Arithmetic mean ± SD
Women	576 (50.2%)
Men	572 (49.8%)
20s	184 (16.0%)
30s	227 (19.8%)
40s	284 (24.7%)
50s	219 (19.1%)
60s	234 (20.4%)
Company employees etc.	520 (45.3%)
Self-employed etc.	88 (7.7%)
Other	540 (47.0%)
Absence of spouse	443 (38.6%)
Presence of spouse	705 (61.4%)
Absence of children	521 (45.4%)
Presence of children	627 (54.6%)
Absence of grandchildren	1001 (87.2%)
Presence of grandchildren	147 (12.8%)
Not pregnant	1125 (98.0%)
Pregnant	23 (2.0%)
Junior or high-school graduate	387 (33.7%)
University graduate, etc.	761 (66.3%)
Science course	339 (29.5%)
Neither	214 (18.6%)
Humanities course	595 (51.8%)
Consumption frequency of fish and shellfish (times/week)	2.66 ± 1.82
Consumption frequency of tuna-type fish(times/week)	0.78 ± 0.92
Thinks it is good for health (health-view)	4.13 ± 0.66
Thinks it is delicious (taste-view)	4.14 ± 0.81
Thinks it can be purchased at an affordable price (availability-view)	3.14 ± 0.96
Does not know consumption guidelines	791 (68.9%)
Knows consumption guidelines	357 (31.1%)

We then obtained data regarding risk perception of mercury in fish and shellfish based on previous studies [22,23], using a 5-point Likert scale ranging from 5: *strongly agree* to 1: *do not agree at all* (Tables 2 and 3). To avoid order effects, these question items were displayed randomly for each respondent.

**Table 2:** Arithmetic mean, standard deviation (SD), and standardized coefficients in confirmatory factor analysis for Slovic’s risk perception model. χ^2^ = 428.68, df = 43, *p* < 0.01; *GFI* = 0.932, *AGFI* = 0.895, *CFI* = 0.875, *RMSEA* = 0.088.

	Arithmetic mean	SD	Standardized coefficients	
			Dread risk	Unknown risk
Cancer risk will increase.	3.25	0.83	0.69	-
Effects on future generations will occur.	3.58	0.87	0.69	-
There may be a fatal effect on health.	3.48	0.91	0.68	-
It is instinctively dreaded.	3.48	0.94	0.67	-
It kills many people at once.	3.10	0.92	0.67	-
Health effects are increasing recently.	3.13	0.81	0.57	-
It is difficult to reduce the health effects.	3.29	0.79	0.50	-
Health effects are immediate. (reversed)	3.32	0.86	-	0.57
Health effects are known to science. (reversed)	2.81	0.83	-	0.51
People surrounding you have correct knowledge about health effects of mercury in fish and shellfish. (reversed)	3.64	0.95	-	0.41
Health effects are unknown.	3.43	0.90	-	-0.06

**Table 3:** Arithmetic mean, standard deviation (SD), and factor pattern matrix for Niiyama’s risk perception question items, and their interpretation. KMO: 0.690, *p* < 0.01 (Bartlett). Bold font: > 0.30 or < -0.30. Cronbach’s α for three and four representative items in Factor 1 and Factor 2 was 0.733 and 0.608, respectively.

	Arithmetic mean	SD	Factor 1	Factor 2
You can trust the government’s regulatory measures for risk reduction.	2.75	0.90	**0.80**	-0.05
You can trust companies and markets for risk reduction.	2.87	0.80	**0.69**	-0.04
You can trust experts’ judgments for risk reduction.	3.09	0.77	**0.59**	0.10
You have heard and read about it.	3.28	0.95	-0.06	**0.64**
Vivid scenes and frightening images of adverse impacts come to mind.	3.20	0.98	-0.03	**0.58**
It is frequently reported in newspapers and television.	3.02	0.91	0.13	**0.52**
The word itself has a negative image.	3.65	0.86	0.00	**0.38**
Interpretations of factors			Trust	Negative impression through information

Subsequently, we presented information concerning consumption guidelines of fish and shellfish for pregnant women:

“While fish and shellfish generally contain nutrients that are healthy, they contain trace amounts of naturally occurring mercury. There are reports that ingestion of mercury by pregnant women (both currently pregnant or those who may be pregnant) may adversely affect the fetus. In Japan, guidelines are given to pregnant women to limit the number of times they consume some specific types of fish and shellfish.”

We asked participants whether they knew this information, since an association between knowledge and risk perception has been previously reported [24–26]. Subsequently, respondents were divided into 3 groups: A1, A2, and A3 and received the following common information:

“In Japan, regulation values are set for mercury in fish and shellfish. Mercury exists not only from artificial origin but also naturally, and regulation values are determined without distinction between artificial and natural mercury. Some specific types of fish and shellfish contain trace amounts of naturally derived mercury that is higher than regulation values. These types of fish and shellfish are excluded from these regulations. As stated previously, the government gives guidelines for pregnant women to limit the number of times they eat some of these types of fish and shellfish.”

We also presented current Japanese guidelines for pregnant women on limiting the consumption amount of fish and shellfish [18]: ≤ once in 2 months, bottlenose dolphin; ≤ once in 2 weeks, short-finned pilot whale; ≤ once per week, alfonsin, swordfish, bluefin tuna, big-eye tuna, finely striate buccinum, Baird's beaked whale, and sperm whale; ≤ twice per week, yellowback seabream, striped marlin, rockfish, southern bluefin tuna, blue shark, Dall’s porpoise, and *Scombrops gilberti*.

In addition, we manipulated the presentation of information on regulation values [17]. For the A1 group, we did not explain the basis of regulation values. For the A2 group, we provided information that the “regulation values of mercury in fish and shellfish (0.4 mg mercury per 1 kg of fish and shellfish) was determined mainly based on findings of epidemiological studies targeting people.” For the A3 group, we provided information that “regulation values of mercury in fish and shellfish (0.4 mg mercury per 1 kg of fish and shellfish) is mainly based on an amount that is 10 times lower than the lowest level of mercury, where adverse effects on health have been observed in epidemiological studies targeting people.” Based on this information, respondents answered a survey regarding their sense of safety about regulation values using a 5-point Likert scale (5: *very safe* to 1: *not safe at all*). Women who were not currently pregnant were asked, “If you were pregnant, how often could you eat tuna-type fish in total (i.e., tuna, big-eye tuna, and bonito) without concern?” (8 choices: *nearly every day*, *5–6 times per week*, *4 times per week*, *3 times per week*, *twice per week*, *once per week*, *less than once per week*, and *do not eat*). The data of each participant are shown in S1 Table.

### Data processing

Occupation, educational background, and science- and humanities-courses were grouped following a previous study [10]. Occupation comprised 3 groups: company employees etc. (e.g., company employees, civil servants, non-profit-organization employees, teachers, lecturers, health professionals, and other professionals); self-employees etc. (e.g., agriculturist, forestry workers, fishery workers, and other self-employed workers); and other (e.g., part-time or casual workers, working on the side, housewives, househusbands, university students, graduate school students, technical college students, junior college students, preparatory school students, jobless, retired, and other). Educational background comprised 2 groups: graduated from junior high school or high school and graduated from a university, etc. (or graduated from an educational facility higher than a junior high school or high school). Science/humanities courses comprised 3 groups: “science course” and “science course chosen from between science course and humanities courses;” “neither;” and “humanities course chosen from between science course and humanities course” and “humanities course.”

Current consumption frequency for fish and shellfish and tuna-type fish were classified as follows: 7 for *nearly every day*, 5.5 for *5–6 times per week*, 4 for *4 times per week*, 3 for *3 times per week*, 2 for *twice per week*, 1 for *once per week*, 0.5 for *less than once per week*, and 0 for *do not eat*.

### Statistical analysis

To evaluate risk perception of mercury in fish and shellfish, we separately conducted confirmatory factor analyses using Slovic’s [22] and Niiyama’s [23] question items. For each item, no ceiling or floor effect was found. The confirmatory factor analysis for Slovic’s model was judged to be reliable (see details in “Factors of Risk Perception of Mercury”, Table 2). However, a reliable model was not created using Niiyama’s question items: *GFI* = 0.921, *AGFI* = 0.830, *CFI* = 0.720, *RMSEA* = 0.157. Therefore, we performed an exploratory factor analysis based on Niiyama’s question items using the maximum-likelihood method and Promax rotation. We extracted two factors based on a parallel analysis [27], scree test, and Kaiser-Guttman method (Table 3 and S1 Fig). The Kaiser-Meyer-Olkin (KMO) measure of sampling adequacy was 0.690. Bartlett’s test was *p* < 0.01. Cronbach’s α for three and four representative items in Factor 1 and Factor 2 was 0.733 and 0.608, respectively. Therefore, the result of the factor analysis was considered reliable.

The difference in factor scores for risk perception among individual attributes were determined by a *t-*test for 2 groups and an analysis of variance using Tukey-Kramer as a post-hoc test for 3 or more. To demonstrate a causal relationship between risk perception and consumption frequency or general beliefs regarding fish and shellfish, Pearson’s correlation coefficients were first estimated among factor scores and between factor scores and current consumption frequency for fish and shellfish and tuna-type fish and beliefs regarding fish and shellfish. After we confirmed the causal relationship between them based on the results (see details in “Discussion”), a multiple regression analysis was conducted with personal attributes including consumption frequency or general beliefs regarding fish and shellfish and knowledge of consumption guidelines (whether the participant knew consumption guidelines or not) as explanatory variables and factor scores for risk perception as objective variables to extract primary factors of risk perception. Through a stepwise regression model, significant variables (*p* < 0.05) were added and insignificant variables (*p* > 0.10) were removed.

A logistic regression analysis was performed to evaluate the impact of information on regulation values and primary risk perception factors on the sense of safety regarding regulation values for fish and shellfish and food-consumption intention. The analysis of sense of safety involved a multivariate ordered logistic regression analysis. For the analysis on food-consumption intention (i.e., excessive avoidance and excessive intake), a binomial logistic regression analysis was used. Considering consumption guidelines for pregnant women in Japan, we used response results for tuna-type consumption frequency if the participant were pregnant, and set excessive avoidance at < once per week and excessive intake at ≥ 3 times per week. Regarding explanatory variables, in addition to Model 1 (using only information on regulation values), the Model 2 analysis was performed using information on regulation values and variables that showed significant association in the multiple regression analysis described above.

Since the group with high tuna-type consumption frequency (≥ once per week) is assumed to have a high risk of mercury exposure, the above analysis targeted not only all respondents (i.e., women who are not currently pregnant in the analysis with excessive avoidance or excessive intake as outcomes), but also the group with current high tuna-type consumption frequency. In the multiple regression and logistic regression analyses, we created dummy variables for each individual attribute (except current consumption frequency for fish and shellfish and tuna-type fish and beliefs regarding fish and shellfish), knowledge of consumption guidelines, and information on regulation values. All variance inflation factors of explanatory variables used in the multiple regression and logistic regression analyses were 2.71 or less. Since they were less than 10, multicollinearity was not a concern. IBM SPSS Version 22, 24 and Amos version 24 were used for the analysis.

## Results

### Factors of risk perception of mercury

In this study, the confirmatory factor analysis for Slocvic’s [22] model was performed to obtain “dread risk” and “unknown risk” of mercury in fish and shellfish (Table 2). Except one item “Health effects are unknown” that had a low standardized coefficient, a reliable model was obtained in general: χ^2^ = 428.68, df = 43, *p* < 0.01; *GFI* = 0.932, *AGFI* = 0.895, *CFI* = 0.875, *RMSEA* = 0.088. The arithmetic mean ± standard deviation (SD) of factors scores in dread and unknown risk were 0 ± 0.92 and 0 ± 0.79, respectively. Two factors were extracted for risk perception using Niiyama’s [23] items (Table 3). Factor 1 was interpreted as items related to trust and factor 2 was interpreted as items related to information and negative impression. Factor 1 was similar to a previous study [23]; however, factor 2 was newly created. Factor 2 was therefore named as “negative impression through information.” The arithmetic mean ± SD of factor scores for trust and negative impression through information were 0 ± 0.87 and 0 ± 0.80, respectively. Significant negative correlations for factor scores were observed between unknown risk and other risk perception, whereas there were significant positive correlations among dread risk, trust, and negative impression through information (S2 Table).

**Fig 1:**
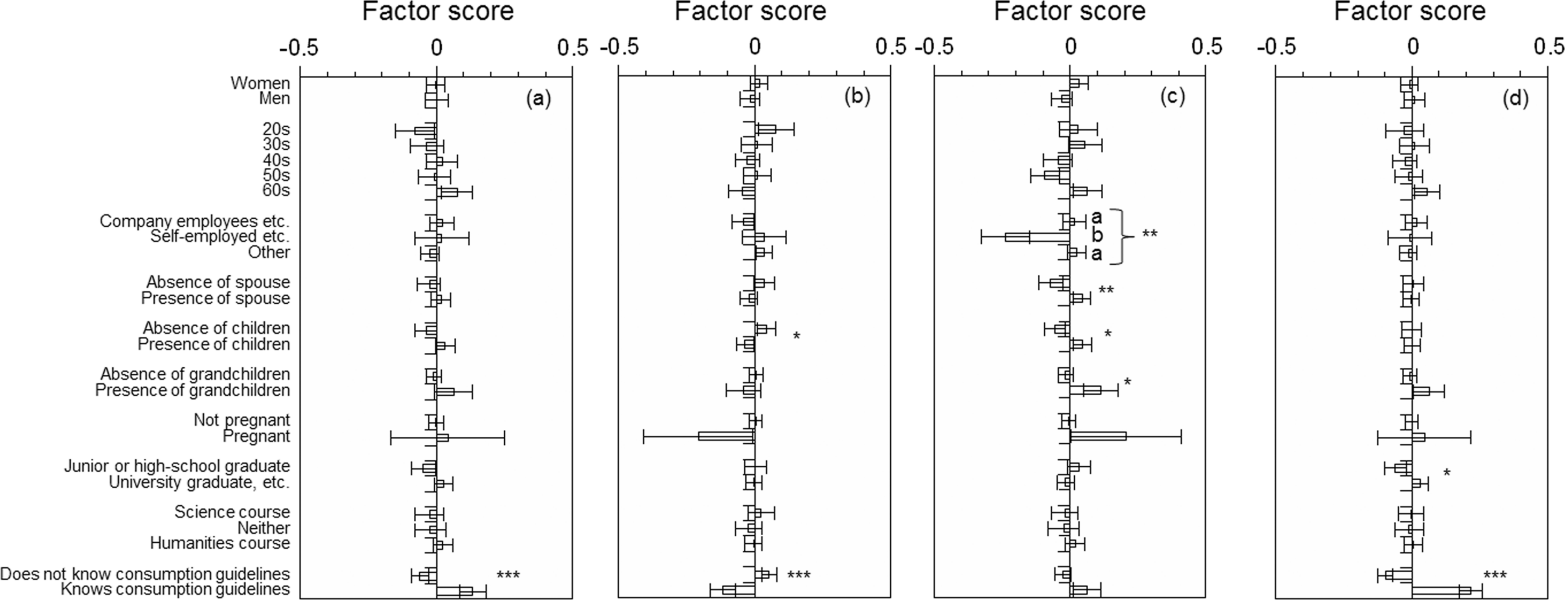
**Factor scores for (a) dread risk, (b) unknown risk, (c) trust, and (d) negative impression through information.** Error bars represent standard errors. *p < 0.10; **p < 0.05; ***p < 0.01. Different letters represent difference among groups upon further analysis (*p* < 0.05).

We examined the relationship between each factor score and individual attributes and knowledge of consumption guidelines (Fig 1 and Table 4). The factor score for dread risk perception was significantly higher among people who consumed tuna-type fish and had high health-view. Those with knowledge of consumption guidelines also had significantly higher dread risk perception. Factor scores for “unknown risk,” were significantly lower in presence of children and knowledge of consumption guidelines. Significant negative correlations were also found with consumption frequency for fish and shellfish and tuna-type fish and availability-view. Trust perception was significantly higher for people with spouses, children, and grandchildren, and lower for self-employed people. They were also significantly higher for people who frequently consumed fish and shellfish or tuna-type fish and had high health-, taste- and availability-views. Negative impression through information was significantly higher among people with a high educational background and knowledge of consumption guidelines. Similar with trust perception, significant positive correlations were found with consumption frequency for fish and shellfish and tuna-type fish and health-, taste- and availability-views.

**Table 4:** Pearson’s correlation coefficient between current consumption frequency for fish and shellfish and tuna-type fish, beliefs about fish and shellfish and factor scores.

	Dread risk	Unknown risk	Trust	Negative impression through information
Consumption frequency of fish and shellfish	0.05	-0.06*	0.10***	0.10***
Consumption frequency of tuna-type fish	0.08***	-0.16***	0.18***	0.12***
Health-view	0.08***	-0.03	0.07**	0.11***
Taste-view	0.02	-0.01	0.07**	0.11***
Availability-view	0.04	-0.11***	0.18***	0.12***

*p < 0.10

**p < 0.05

***p < 0.01.

To evaluate primary factors related to risk perception of mercury, we performed multiple regression analysis among individual attributes and knowledge of consumption guidelines (Table 5). Tuna-type consumption frequency, health-view and knowledge of consumption guidelines showed significant positive associations with dread risk perception. For unknown risk perception, tuna-type consumption frequency, availability-view, and knowledge of consumption guidelines showed negative associations. Self-employed etc., presence of spouse, tuna-type consumption frequency, and availability-view were significantly associated with trust. For negative impression through information, tuna-type consumption, health- and availability-views, and knowledge of consumption guidelines showed significant associations. These were identified as primary factors governing risk perception of mercury.

**Table 5:** Regression coefficients for risk perception regarding mercury in fish and shellfish. B: unstandardized regression coefficient; CI: confidence interval; β: standardized partial coefficient; Ref: reference. No significant coefficients were obtained for sex, age, presence/absence of children, grandchildren, and pregnancy, science/humanities courses, consumption frequency of fish and shellfish, and taste-view.

	Dread risk			Unknown risk			Trust			Negative impression through information		
	B (95% CI)	β		B (95% CI)	β		B (95% CI)	β		B (95% CI)	β	
Constant	-0.51 (-0.85–-0.17)	-	***	0.37 (0.21–0.52)	-	***	-0.57 (-0.75–-0.39)	-	***	-0.82 (-1.13–-0.51)	-	***
Company employees etc. = Ref	-	-	-	-	-	-	-	-	-	-	-	-
Self-employed etc.	-	-	-	-	-	-	-0.24 (-0.42–-0.05)	-0.07	**	-	-	-
Other	-	-	-	-	-	-	-	-	-	-	-	-
Absence of spouse = Ref	-	-	-	-	-	-	-	-	-	-	-	-
Presence of spouse	-	-	-	-	-	-	0.11 (0.01–0.21)	0.06	**	-	-	-
Tuna-type consumption frequency	0.06 (0.01–0.12)	0.06	**	-0.12 (-0.17–-0.06)	-0.13	***	0.14 (0.09–0.20)	0.15	***	0.07 (0.02–0.12)	0.08	***
Health-view	0.10 (0.02–0.18)	0.07	**							0.10 (0.03–0.17)	0.09	***
Availability-view	-	-	-	-0.07 (-0.12–-0.02)	-0.09	***	0.13 (0.08–0.18)	0.14	***	0.08 (0.03–0.13)	0.09	***
Does not know consumption guidelines = Ref	-	-	-	-	-	-	-	-	-	-	-	-
Knows consumption guidelines	0.18 (0.07–0.30)	0.09	***	-0.16 (-0.25–-0.06)	-0.09	***	-	-	-	0.31 (0.21–0.40)	0.18	***

*p < 0.10

**p < 0.05

***p < 0.01

ns: not significant.

### Effects of information presentation of the basis of regulation values and primary factors of risk perception on sense of safety and food-consumption intentions

Regarding sense of safety about mercury regulations for fish and shellfish, 2.5% believed it to be very safe, 28.7% as somewhat safe, 60.4% as neither safe nor unsafe, 7.1% as somewhat unsafe, and 1.3% as not all safe,. Overall,429 (75.9%) out of 565 participants (who were currently not pregnant women) demonstrated excessive avoidance. Similarly, the percentage of excessive intake was 4.2%.Women who believed that they would eat tuna-type fish ≥ 3 times per week without concern if they were pregnant were minor.

We evaluated the effect of information presentation of the basis of regulation values and the primary factors of risk perception (as mentioned in “Factors of Risk Perception of Mercury”) on sense of safety regarding mercury regulations for fish and shellfish as well as tuna-type consumption intentions using a logistic regression analysis (Table 6). When we targeted all respondents, significant associations were observed between sense of safety and presence of spouse, health- and availability-views toward fish and shellfish, and knowledge of consumption guidelines. This adjusted odds ratio (OR; 95% confidence interval [CI]) was 0.73 (0.57–0.93), 1.99 (1.65–2.39), 1.15 (1.02–1.30), and 1.64 (1.27–2.11), respectively (Model 2). No significant association was seen in either model between sense of safety and information presentation on regulation values.

**Table 6:** Associations between objective variables—sense of safety, excessive avoidance, and excessive intake—and explanatory variables—information on regulation values, individual attributes, and knowledge. For excessive avoidance and excessive intake, women who were not pregnant were targeted. OR: odds ratio; CI: confidence interval; Ref: reference.

	Whole						High tuna-type consumption group					
	Sense of safety		Excessive avoidance		Excessive intake		Sense of safety		Excessive avoidance		Excessive intake	
	OR (95%CI)		OR (95%CI)		OR (95%CI)		OR (95%CI)		OR (95%CI)		OR (95%CI)	
Model 1												
Information on regulation values (A1) = Ref	1		1		1		1		1		1	
A2	1.09 (0.82–1.45)	ns	1.04 (0.65–1.66)	ns	1.99 (0.59–6.72)	ns	1.27 (0.73–2.20)	ns	1.04 (0.46–2.36)	ns	1.57 (0.37–6.72)	ns
A3	1.15 (0.86–1.53)	ns	1.14 (0.71–1.84)	ns	2.95 (0.93–9.32)	*	2.04 (1.18–3.53)	**	1.75 (0.76–4.01)	ns	2.10 (0.52–8.47)	ns
Model 2												
Information on regulation values (A1) = Ref	1		1		1		1		1		1	
A2	1.08 (0.81–1.44)	ns	1.14 (0.69–1.87)	ns	1.77 (0.48–6.60)	ns	1.32 (0.75–2.31)	ns	1.07 (0.44–2.59)	ns	1.67 (0.33–8.34)	ns
A3	1.06 (0.80–1.42)	ns	1.32 (0.80–2.17)	ns	2.69 (0.78–9.22)	ns	2.04 (1.16–3.57)	**	1.75 (0.73–4.18)	ns	2.47 (0.55–11.2)	ns
Company employees etc. = Ref	1		1		1		1		1		1	
Self-employed etc.	0.95 (0.60–1.50)	ns	0.72 (0.26–1.95)	ns	2.61 (0.44–15.30)	ns	1.00 (0.41–2.48)	ns	0.35 (0.04–2.79)	ns	3.21 (0.17–60.9)	ns
Other	1.21 (0.95–1.54)	ns	1.09 (0.67–1.77)	ns	0.80 (0.26–2.46)	ns	0.94 (0.59–1.49)	ns	1.12 (0.46–2.73)	ns	0.94 (0.21–4.22)	ns
Absence of spouse = Ref	1		1		1		1		1		1	
Presence of spouse	0.73 (0.57–0.93)	**	1.09 (0.69–1.72)	ns	0.94 (0.34–2.58)	ns	0.63 (0.39–1.01)	*	2.34 (1.03–5.34)	**	0.59 (0.15–2.24)	ns
Tuna-type consumption frequency	1.07 (0.94–1.22)	ns	0.51 (0.39–0.66)	***	2.06 (1.54–2.76)	***	0.97 (0.82–1.15)	ns	0.81 (0.59–1.11)	ns	1.68 (1.16–2.43)	***
Health-view	1.99 (1.65–2.39)	***	0.93 (0.66–1.31)	ns	0.78 (0.36–1.71)	ns	1.56 (1.09–2.23)	**	1.05 (0.55–2.00)	ns	0.46 (0.15–1.44)	ns
Availability-view	1.15 (1.02–1.30)	**	0.83 (0.67–1.04)	*	2.08 (1.21–3.55)	***	1.10 (0.86–1.41)	ns	0.73 (0.49–1.08)	ns	2.50 (1.18–5.29)	**
Does not know consumption guidelines = Ref	1		1		1		1		1		1	
Knows consumption guidelines	1.64 (1.27–2.11)	***	1.21 (0.77–1.90)	ns	1.08 (0.41–2.83)	ns	1.44 (0.90–2.29)	ns	1.07 (0.51–2.21)	ns	1.60 (0.48–5.28)	ns

*p < 0.10

**p < 0.05

***p < 0.01

ns: not significant.

For excessive avoidance, significant negative associations were observed with tuna-type consumption frequency: adjusted OR (95% CI) was 0.51 (0.39–0.66) (Model 2). Availability-view also showed a weak but significant association: 0.83 (0.67–1.04). For excessive avoidance, no significant association was seen with presenting information on regulation values in either model. For excessive intake, significant positive associations were seen for tuna-type consumption frequency and availability-view: adjusted OR (95% CI) was 2.06 (1.54–2.76) and 2.08 (1.21–3.55), respectively (Model 2). In Model 1, a weak but significant positive association was found when presenting the basis of regulatory values (A3 group) (*p* < 0.10, where adjusted OR (95% CI) was 2.95 (0.93–9.32)). Although no significant association was seen in Model 2, positive directivities were observed for excessive intake with presenting information on regulation values: adjusted ORs (95% CI) were 1.77 (0.48–6.60) for A2 and 2.69 (0.78–9.22) for A3.

When targeting groups that ingest tuna at a high frequency, in both models, significant positive associations were seen between sense of safety and information presentation based on regulation values (A3) in terms of adjusted ORs (95% CI): 2.04 (1.18–3.53) for Model 1 and 2.04 (1.16–3.57) for Model 2. In Model 2, health-view showed a significantly positive association: 1.56 (1.09–2.23). People with a spouse had a weak but significantly lower sense of safety (*p* < 0.10, adjusted OR (95% CI): 0.63 (0.39–1.01) for Model 2). Regarding excessive avoidance, a significant positive association with presence of spouse was observed in terms of adjusted OR (95% CI): 2.34 (1.03–5.34) for Model 2. No significant association was observed in either model for presentation of information on regulation values. For excessive intake, a significant positive association was observed with tuna-type consumption frequency and availability-view (adjusted ORs (95% CI): 1.68 (1.16–2.43) and 2.50 (1.18–5.29), respectively; Model 2). Information on regulation values was not significantly associated with these consumption behaviors in either model.

## Discussion

Among individual attributes and knowledge, there was a strong relationship between risk perception of mercury and tuna-type consumption frequency, health- and availability-views, knowledge of consumption guidelines, presence of spouse, and job-type. In particular, tuna-type consumption frequency was significantly associated with all four perceptions through bivariate correlation analysis (Table 4): individuals who frequently consumed tuna felt that there was a dread risk, perceived that the risks were well known, trusted regulatory measures, and had negative impressions through information sources. These results reflect a causal relationship between risk perception and consumption frequency. If high risk perception affects consumption frequency, the correlation should be negative (i.e., people do not eat tuna because of the high risk perception). However, while there was a negative correlation between unknown risk perception and tuna-type consumption frequency, a positive correlation between dread risk perception and tuna-type consumption frequency was observed. It is an unreasonable interpretation that people eat certain types of tuna because they experience dread. This result suggests a contrary causal effect: consumption frequency affected dread risk perception (i.e., people feel that consuming tuna-type fish involves dread risk because people already consume tuna). Similar results were found between mercury risk perceptions and general beliefs regarding fish and shellfish. It is thought that general beliefs such as health- and availability-views toward fish and shellfish form impressions about fish and shellfish themselves, and that these create mercury risk perception in hindsight. Multiple regression analysis based this causal relationship between risk perception and consumption frequency or general beliefs regarding fish and shellfish also showed results consistent with a bivariate correlation analysis and highlighted that consumption frequency was a primary factor of risk perceptions (Table 5). In brief, these results imply that dread risk perception of mercury does not govern tuna-type consumption behavior, but rather individuals’ consumption behavior governs dread risk perception of mercury. When social attention to a phenomenon increases, such as radionuclides after a nuclear power plant accident, excessive food avoidance can occur due to high risk perception [28,29]. However, mercury risks in fish and shellfish are currently “out of sight, out of mind” for many Japanese people, and it is believed that these do not contribute to consumption behavior.

The results also showed strong correlations between mercury risk perceptions and knowledge of consumption guidelines. Interestingly, while knowledge of consumption guidelines strengthens dread risk perception, it weakens perceptions about unknown risks. It makes sense that knowledge weakens the perception of unknown risks. Previous studies have yielded inconsistent results where knowledge has been found to increase and decrease risk perception [24–26]; however, results obtained in this study show that knowledge impacts risk perception in different ways depending on the kind of risk perception.

The logistic analysis results found that people with higher tuna-type consumption frequency did not avoid tuna-type fish as much, and easily fell into the excessive-intake category. The people positive on health- and availability-views toward fish and shellfish had a higher sense of safety with regard to regulatory measures. Excessive intake was high in the high availability-view group. These results show that sense of safety in regulatory measures is related to one’s value of fish and shellfish, from a health and availability perspective and consumption frequency. This is consistent with its association with risk perception. While people with high current tuna-type consumption frequency or greater access to fish and shellfish are considered as the mercury high-risk group, they are likely to have excessive intake, even when pregnant.

Sense of safety differed by presentation of information on regulation values in the group with high tuna-type consumption. The A3 group had a significantly higher sense of safety compared to the A1 group (OR (95%CI): 2.04 (1.18–3.53)). This suggests that presenting information on regulation values increases the sense of safety among those with high tuna-type consumption. Since the group with high tuna-type consumption is equivalent to a mercury high-risk group and has strong interest in their own mercury risks, it is inferred that this knowledge regarding a “10 times” safety factor makes them perceive regulatory measures as safe. Those with knowledge of consumption guidelines had a significantly and strongly higher sense of safety (for all respondents; OR (95%CI): 1.64 (1.27–2.11)), suggesting that acquiring knowledge has a potential positive impact on developing a sense of safety.

However, information on regulation values showed a weak but significant positive association with excessive intake. Although this information enhances sense of safety, there is also danger of causing excessive intake.

In this study, the authors did not intend to determine the appropriateness of the types of presentation methods or information. People’s perceptions and decision-making are influenced by a framing effect [30]. Therefore, information providers and authorities should design ethically justified methods for information presentation and risk communication, after considering the impact of the information presented [31].

A lacking of a sense of safety (e.g., anxiety) may positively affect risk avoidance; however, it also has negative impacts such as decreased well-being, risk tradeoff (e.g., diet with poor nutritional balance, and economic loss in specific industries), and psychological distress [2–4,32,33]. Excessive distress potentially causes increased mortality via suicide and cancer [34–36]. Information providers and authorities must aim to alleviate excessive anxiety and improve well-being for the public by promoting a sense of safety. They must also simultaneously reduce risks such as excessive intake. In the case of mercury regulation, information providers and authorities should explain consumption limits and their risks more carefully, especially with regard to deterring excessive intake among high-risk groups.

### Limitations and future perspectives

This study had some limitations. First, there were potential participant biases. We conducted an online survey through which biases may have been introduced. However, since respondents acquired points through online surveys, respondents with no interest in this survey’s topic may have been motivated to participate; this is potentially more effective for reducing bias than central-location testing or mailing methods. To reduce bias in this study, factors such as individual attributes were categorized and the strength of their associations was examined. The three presentation conditions for information on regulation values, which is this study’s focus, were randomly assigned to participants. Second, this study dealt with mercury risks in fish and shellfish and presenting information on regulation values; however, we did not discuss generalizations regarding other risks and regulations. In particular, with the spread of Minamata disease, Japan has historically experienced pollution-related problems; therefore, it is possible that mercury risk perception and sense of safety differs in Japan and other countries.

Despite these limitations, this study revealed that dread risk perception of mercury did not govern tuna-type consumption behavior; rather, individuals’ consumption behavior governed dread risk perception of mercury. Although presenting information on regulation values increases the high-risk group’s sense of safety, there is a danger of promoting increased risk associated with excessive intake. Future research can deepen our understanding on presenting other information on regulation values. This can help information providers and authorities support individuals’ decision-making and reduce societal risks, ultimately leading to ethical, justifiable, and appropriate information presentation and risk communication methods.

## Supporting information

S1 Fig Scree plots of factor analysis regarding Niiyama’s risk perception question items. Scree plots in parallel analysis are also shown.
(DOCX)

S1 Table Data of each participant. (XLSX)

S2 Table Pearson’s correlation coefficients in factor scores among individual risk perceptions. (DOCX)

## References

[1] Murakami M (2016) Risk analysis as regulatory science: Toward the establishment of standards. Radiat Prot Dosim 171: 156–162. 10.1093/rpd/ncw211 PMC5035387 27475751

[2] Kuttschreuter M (2006) Psychological determinants of reactions to food risk messages. Risk Anal 26: 1045–1057. doi: 10.1111/j.1539-6924.2006.00799.x 16948696 10.1111/j.1539-6924.2006.00799.x

[3] Setbon M, Raude J, Fischler C, Flahault A (2005) Risk perception of the "mad cow disease" in France: Determinants and consequences. Risk Anal 25: 813–826. doi: 10.1111/j.1539-6924.2005.00634.x 16268931 10.1111/j.1539-6924.2005.00634.x

[4] Hommerich C (2012) Trust and subjective well-being after the Great East Japan Earthquake, tsunami and nuclear meltdown: Preliminary results. Int J Jpn Sociol 21: 46–64.

[5] National Research Council (1987) Improving risk communication. Washington D.C., National Academy Press.

[6] Food and Agriculture Organization of the United Nations, World Health Organization (2016) Risk communication applied to food safety handbook: http://www.fao.org/3/a-i5863e.pdf. Accessed: 31 Jan. 2016

[7] Covello VT, Sandman PM, Slovic P (1988) Risk communication, risk statistics, and risk comparisons: A manual for plant managers. Washington, DC: Chemical Manufacturers Association.

[8] Roth E, Morgan MG, Fischhoff B, Lave L, Bostrom A (1990) What do we know about making risk comparisons? Risk Anal 10: 375–387.

[9] Hino Y, Murakami M, Midorikawa S, Ohtsuru A, Suzuki S, Tsuboi K, et al (2016) Explanatory meetings on thyroid examination for the "Fukushima Health Management Survey" after the Great East Japan Earthquake: Reduction of anxiety and improvement of comprehension, and satisfaction. Tohoku J Exp Med 239: 333–343. doi: 10.1620/tjem.239.333 27535010 10.1620/tjem.239.333

[10] Murakami M, Nakatani J, Oki T (2016) Evaluation of risk perception and risk-comparison information regarding dietary radionuclides after the 2011 Fukushima nuclear power plant accident. PLos One 11: e0165594 doi: 10.1371/journal.pone.0165594 27802304 10.1371/journal.pone.0165594 PMC5089555

[11] Visschers VHM, Siegrist M (2013) How a nuclear power plant accident influences acceptance of nuclear power: Results of a longitudinal study before and after the Fukushima Disaster. Risk Anal 33: 333–347. doi: 10.1111/j.1539-6924.2012.01861.x 22762151 10.1111/j.1539-6924.2012.01861.x

[12] Sjoberg L (2000) Factors in risk perception. Risk Anal 20: 1–11. 10795334

[13] Kahan DM, Braman D, Gastil J, Slovic P, Mertz CK (2007) Culture and identity-protective congnition: Explaining the while-male effect in risk perception. J Empir Legal Stud 4: 465–505.

[14] The Food Safety Commission Japan The Contaminant Expert Committee (2005) Food safety risk assessment related to methylmercury in seafood: http://www.fsc.go.jp/sonota/methylmercury_risk_assessment.pdf. Accessed: 31 Jan. 2017

[15] Ha E, Basu N, Bose-O'Reilly S, Dorea JG, McSorley E, Sakamoto M, et al (2017) Current progress on understanding the impact of mercury on human health. Environ Res 152: 419–433. doi: 10.1016/j.envres.2016.06.042 27444821 10.1016/j.envres.2016.06.042

[16] Sheehan MC, Burke TA, Navas-Acien A, Breysse PN, McGready J, Fox MA (2014) Global methylmercury exposure from seafood consumption and risk of developmental neurotoxicity: A systematic review. Bull World Health Organ 92: 254–269f. doi: 10.2471/BLT.12.116152 24700993 10.2471/BLT.12.116152 PMC3967569

[17] Ministry of Health Labour and Welfare (1973) Provisional regulations on mercury in fishery products: http://www.mhlw.go.jp/shingi/2003/06/dl/s0603-4a.pdf. Accessed: 31 Jan. 2017 [in Japanese]

[18] Ministry of Health Labour and Welfare (2010) Mercury in fishery products: http://www.mhlw.go.jp/topics/bukyoku/iyaku/syoku-anzen/suigin/index.html. Accessed: 31 Jan. 2017 [in Japanese]

[19] U.S. Food & Drug Administration (2017) Eating fish: What pregnant women and parents should know: http://www.fda.gov/Food/FoodborneIllnessContaminants/Metals/ucm393070.htm. Accessed: 31 Jan. 2017

[20] Karimi R, Silbernagel S, Fisher NS, Meliker JR (2014) Elevated blood Hg at recommended seafood consumption rates in adult seafood consumers. Int J Hyg Environ Health 217: 758–764. doi: 10.1016/j.ijheh.2014.03.007 24780236 10.1016/j.ijheh.2014.03.007

[21] Oki S, Nakayachi K (2012) Paradoxical effects of the record-high Tsunamis caused by the 2011 Tohoku Earthquake on public judgments of danger. Int J Disaster Risk Reduction 2: 37–45.

[22] Slovic P (1987) Perception of risk. Science 236: 280–285. 3563507 10.1126/science.3563507

[23] Niiyama Y, Kito Y, Hosono H, Kawamura R, Kudo H, Kiyohara A (2011) The structural models of public risk perception of typical food-related hazards: An analysis of the structural complexity of incorporated factors by SEM. Jpn J Risk Anal 21: 295–306. [in Japanese]

[24] Vandermoere F (2008) Hazard perception, risk perception, and the need for decontamination by residents exposed to soil pollution: The role of sustainability and the limits of expert knowledge. Risk Anal 28: 387–398. doi: 10.1111/j.1539-6924.2008.01025.x 18419656 10.1111/j.1539-6924.2008.01025.x

[25] Perko T, Zeleznik N, Turcanu C, Thijssen P (2012) Is knowledge important? Empirical research on nuclear risk communication in two countries. Health Phys 102: 614–625. doi: 10.1097/HP.0b013e31823fb5a5 22570920 10.1097/HP.0b013e31823fb5a5

[26] Imai H, Okumiya K, Fukutomi E, Wada T, Ishimoto Y, Kimura Y, et al (2015) Association between risk perception, subjective knowledge, and depression in community-dwelling elderly people in Japan. Psychiatry Res 227: 27–31. doi: 10.1016/j.psychres.2015.03.002 25813776 10.1016/j.psychres.2015.03.002

[27] Hori K (2001) Parallel analysis: http://www.ec.kagawa-u.ac.jp/~hori/delphistat/index.html#pa. Accessed: 14 July, 2017 [in Japanese]

[28] Orita M, Hayashida N, Nakayama Y, Shinkawa T, Urata H, Fukushima Y, et al (2015) Bipolarization of risk perception about the health effects of radiation in residents after the accident at Fukushima nuclear power plant. PLos One 10: e0129227 doi: 10.1371/journal.pone.0129227 26057539 10.1371/journal.pone.0129227 PMC4461282

[29] Hayano RS, Tsubokura M, Miyazaki M, Ozaki A, Shimada Y, Kambe T, et al (2015) Whole-body counter surveys of over 2700 babies and small children in and around Fukushima Prefecture 33 to 49 months after the Fukushima Daiichi NPP accident. Proc Jpn Acad Ser B Phys Biol Sci 91: 440–446. doi: 10.2183/pjab.91.440 26460321 10.2183/pjab.91.440 PMC4729858

[30] McNeil BJ, Pauker SG, Sox HC Jr., Tversky A (1982) On the elicitation of preferences for alternative therapies. N Engl J Med 306: 1259–1262. doi: 10.1056/NEJM198205273062103 7070445 10.1056/NEJM198205273062103

[31] Murakami M, Tsubokura M (2017) Evaluating risk communication after the Fukushima disaster based on nudge theory. Asia-Pac J Public He 29: 193s–200s. 10.1177/1010539517691338 28330399

[32] Gigerenzer G (2006) Out of the frying pan into the fire: Behavioral reactions to terrorist attacks. Risk Anal 26: 347–351. doi: 10.1111/j.1539-6924.2006.00753.x 16573625 10.1111/j.1539-6924.2006.00753.x

[33] Suzuki Y, Yabe H, Yasumura S, Ohira T, Niwa S, Ohtsuru A, et al (2015) Psychological distress and the perception of radiation risks: The Fukushima Health Management Survey. Bull World Health Organ 93: 598–605. doi: 10.2471/BLT.14.146498 26478623 10.2471/BLT.14.146498 PMC4581639

[34] Pratt LA (2009) Serious psychological distress, as measured by the K6, and mortality. Ann Epidemiol 19: 202–209. doi: 10.1016/j.annepidem.2008.12.005 19217003 10.1016/j.annepidem.2008.12.005

[35] Mattisson C, Bogren M, Bradvik L, Horstmann V (2015) Mortality of subjects with mood disorders in the Lundby community cohort: A follow-up over 50 years. J Affect Disord 178: 98–106. doi: 10.1016/j.jad.2015.02.028 25801522 10.1016/j.jad.2015.02.028

[36] Batty GD, Russ TC, Stamatakis E, Kivimaki M (2017) Psychological distress in relation to site specific cancer mortality: Pooling of unpublished data from 16 prospective cohort studies. BMJ 356: j108 doi: 10.1136/bmj.j108 28122812 10.1136/bmj.j108 PMC5266623

