# Supplementary material for: Presenting information on regulation values improves the public’s sense of safety: Perceived mercury risk in fish and shellfish and its effects on consumption intention
Source: PLoS One. 2017 Dec 21;12(12):e0188758. doi: 10.1371/journal.pone.0188758 (PMC5739387; doi:10.1371/journal.pone.0188758)
Supplement: S1 Fig — Scree plots in parallel analysis are also shown. (DOCX) [file pone.0188758.s001.docx]

**S1 Fig. Scree plots of factor analysis regarding Niiyama’s risk perception question items.** Scree plots in parallel analysis are also shown.
