# Supplementary material for: Presenting information on regulation values improves the public’s sense of safety: Perceived mercury risk in fish and shellfish and its effects on consumption intention
Source: PLoS One. 2017 Dec 21;12(12):e0188758. doi: 10.1371/journal.pone.0188758 (PMC5739387; doi:10.1371/journal.pone.0188758)
Supplement: S2 Table — (DOCX) [file pone.0188758.s003.docx]

**S2 Table.**

**Pearson’s correlation coefficients in factor scores among individual risk perceptions.**

|  | Unknown risk | Trust | Negative impression through information |
| --- | --- | --- | --- |
| Dread risk | -0.86*** | 0.16*** | 0.65*** |
| Unknown risk |  | -0.35*** | -0.63*** |
| Trust |  |  | 0.33*** |

* *p* < 0.10, ** *p* < 0.05, *** *p* < 0.01.
